# Q-T interval prolongation in cirrhosis: Relationship and severity

**DOI:** 10.22088/cjim.9.3.239

**Published:** 2018

**Authors:** Ali-Akbar Hajiaghamohammadi, Mohammad-Mahdi Daee, Ali Zargar, Somayeh Ahmadi-Gooraji, Alfred Rahban, Fereshte Attaran

**Affiliations:** 1 Department of Internal Medecine, Metabolic Disease Research Center, Qazvin University of Medical Sciences, Qazvin, Iran; 2 Department of Cardiology, Boali Hospital, Qazvin University of Medical Sciences, Qazvin, Iran; 3 Velayat Clinical Research Development Unit, Qazvin University of Medical Sciences, Qazvin, Iran; 4 Hollywood Presbyterian Medical Center, Midway Hospital Medical Center, Los Angles, California, USA

**Keywords:** Cardiomyopathy, Prolonged QT interval, Severity of cirrhosis, Child score

## Abstract

**Background:**

Cirrhosis as the final stage of progressive fibrosis of liver can affect other organs such as lungs, kidneys and heart. "Cirrhotic cardiomyopathy" involves the electrophysiological abnormalities such as QT interval prolongation. We assessed correlation between corrected QT interval prolongation and severity of cirrhosis based on Child Classification in each ECG lead.

**Methods:**

In this case-control study, the patients attending the outpatient clinic and inpatient department of internal medicine of Velayat Hospital in Qazvin were enrolled from September 2014 to July 2015. Total samples were 74 patients, half of which were used as controls. Cirrhosis severity was determined as per Child Classification. Both groups had Ca^2+^, Mg^2+^, K^+^ tested and 12- lead ECG was obtained. The QT interval was corrected by two different formulas: (1) QTc=QT/√RR (QTc_1_); (2) QTc=QT+1.75 (heart rate-60) (QTc_2_). To analyze the data, the software SPSS Version 16 and Mann-Whitney, Pearson’s chisquare test-Kruskal-Wallis, and t-tests were used.

**Results:**

The mean of QTc_1_ and QTc_2_ was longer in cirrhotics than the control group. There was a significant correlation between Child score and length of QTc_1_ in leads: III (p=0.032), AVL (p=0.041), V2 (p=0.049), V6 (p=0.015). There were significant differences in length of QTc_1_ in leads: V3 (p=0.031) and V6 (p=0.021); and QTc_2_ in lead V3 (p=0.039) between Child Classification.

**Conclusions:**

Cirrhosis can induce QTc interval prolongation. Lead V3 has statistically significant correlation with the severity of cirrhosis based on child classification. We propose that QT interval prolongation be added as a criterion for prioritizing liver transplantation.

Cirrhosis is the final stage of progressive fibrosis of liver which can also affect other organs such as the lungs, kidneys and heart simultaneously by poorly understood interactions. The term of "cirrhotic cardiomyopathy" was first used 20-30 years ago by 'Lee' (1) to point out the cardiac dysfunction in cirrhosis. In 2005, a panel of expert hepatologists and cardiologists met at the World Congress of Gastroentrology and suggested to adopt the definition of cirrhotic cardiomyopathy as a form of chronic cardiac dysfunction in patients with cirrhosis. This would be characterized by reduced contractile responsiveness to stress, and/or altered diastolic relaxation and/or electrophysiological abnormalities in the absence of other known cardiac disease (2). Thus a part of this definition also involves the electrophysiological abnormalities, which can be seen on an electrocardiogram (ECG). One of these ECG findings are the prolongation of QT interval (1). Prolongation of the QT interval reflects prolongation of ventricular repolarization period that may result in fatal arrhythmias (3).

Large studies, like Cardiovascular Health Study, the NHAMES study, the Strong Heart Study, Rotterdom and a recent meta-analysis have shown a correlation between the prolongation of QTc and cardiovascular mortality rate and sudden death in general population as a whole (4), but the relation between the prolonged QTc and cardiovascular events need to be further studied in cirrhotic patients. We believe that cirrhosis can induce prolongation of QT interval and the length of QT interval directly correlates with the severity of cirrhosis that is indicated in the Child Classification and prolonged corrected QT interval can predict a higher risk in these patients. This additional risk of prolonged QT interval potentially can be added to the criteria for prioritization of hepatic transplantation.

In the present study, we assessed the correlation between corrected QT interval prolongation and severity of cirrhosis based on Child Classification in each ECG lead.

## Methods

In this case-control study, the patients attending the outpatient clinic and inpatient department of Internal Medicine of Velayat Hospital in Qazvin, were enrolled from September 2014 to July 2015.Total samples were 74 patients considered as the case (n=37) and control groups (n=37). In cirrhotic group (case), diagnosis was made on the basis of clinical, ultrasonography and histopathology and their severity was determined as per child scoring and classification system. Based on that, 12 patients were in class A, 12 in class B and 13 in class C. Also, on the same time frame, the control group was randomly selected from hospitalized patients at the same hospital, and did not have exclusion criteria. The control group was matched except for those with cirrhosis. In cirrhotics, serum albumin, prothrombin time, INR and total bilirubin were tested to determine their child score. Both groups had Ca^2+^, Mg^2+^, K^+ ^tested.

Exclusion criteria from this study were: Serum calcium (Ca^+2^)<8.5 mg/dl (5) and/or serum potassium (K^+^) <3.6Mm (6) and/or serum magnesium (Mg^2)+^<1.8meq/dl (7), any bundle branch block in ECG, past history of ischemic or non-ischemic heart disease and drug history which could prolong the QT interval such as: macrolides and quinolones, tricyclics and selective serotonin reuptake inhibitors, haloperidol and phenotiazine, ondansetron and prochloperazine (8). After the selection of samples, 12- lead ECG was obtained from both groups (speed was set at 25mm/s and amplitude of 10mm/mV). QT interval was measured manually from the onset of Q wave(or R) to the end of T wave. End of T wave was taken to be where its descending limb returns to baseline. Thereafter, the QT interval was corrected by the following two different formulas;

1. Bazett’s formula or formula#1 as QTc=QT/√RR. Corrected QT interval from this formula is called QTc_1_ in our report.

2. Formula#2 as QTc=QT+1.75 (heart rate-60) (QT as per millisecond). Corrected QT interval from this formula is called QTc_2_ in our report.

QTc longer than 0.45 second in females and 0.44 second in males were considered as prolonged by both formulas (4). To analyze the data, the following software and tests were used: the software SPSS16, Mann-Whitney test, t-test, Pearson-chi-square test and Kruskal-Wallis test. A p-value <0.05 was taken to be statistically significant.

## Results

Data from 37 patients (21 males and 16 females) and 37 controls (24 males and 13 females) were analyzed. Range of age in patients with cirrhosis was between 28 to 84 years old with the mean of 58.75±11.47 years. Range of age in the control group was from 30 to 82 with the mean of 59.15±11.2 years. The two groups were matched according to age and gender (age: p=0.854, gender: p=0.475). The use of Mann-Whitney test showed that in all twelve leads, mean of QTc_1_ and QTc_2_ was longer in cirrhotic patients than that of the control group. The difference in these leads was statistically significant (table 1). By using the Pearson's correlation test, there was a statistically significant correlation between Child score and length of QTc_1_ in leads: III (R: 0.354, p=0.032), AVL (R: 0.338, p=0.041), V2 (R: 0.326, p=0.049), V6 (R: 0.396, p=0.015). When we used Kruskal-Wallis test, there were statistically significant differences between Child Classification and length of QTc_1_ in leads: V3 (p=0.031) and V6 (p=0.021). Finally, by using Pearson [correlation between child score and length of QTc_2_ but by Kruskal-Wallis test, there was one shown in lead V3 (p=0.039). The number of patients with prolonged QTc_1_ was more in cirrhotic group as compared to the control group and this difference was statistically significant in all leads. But by using formula no.2, these differences were only statistically significant in leads: II, aVR, aVF, V3, V4, V5 and V6 (table 2).

**Table 1:** Mean and SD of QTc_1_§ and QTc2 § intervals per lead, in case (n=37) and control groups (n=37)

**Leads**	**QTc**_1_ **,** **mean±SD** **(per milisecond)**			**QTc**_2_ **, ** **mean±SD** **(per milisecond)**		
	**Case **	**Control **	**P-value** *	**Case**	**Control **	**P-value** *
I	411 ± 39	391± 28	0.021	394 ± 34	377±23	0.016
II	420±43	390±30	0.0014	403±38	377±26	0.002
III	406±46	383±26	0.019	389±38	370±22	0.025
aVR	426±47	395±25	0.002	410±37	380±22	0.000
aVL	406±42	385±29	0.048	391±36	372±24	0.023
aVF	420±39	384±30	0.000	401±33	372±26	0.000
V1	410±34	381±34	0.001	394±27	368±28	0.001
V2	421±41	390±28	0.000	403±34	377±23	0.000
V3	427±43	400±29	0.002	407±35	385±24	0.003
V4	431±39	398±28	0.000	412±33	385±24	0.001
V5	423±45	397±28	0.005	408±38	384±26	0.002
V6	421±40	392±29	0.001	405±34	379±26	0.001

* P-value<0.05 is statisticallay significant.

§ QTc_1_ is corrected QT interval that calculated by formula No.1as:QT/√RR. QTc_2_ is corrected QT interval that calculated by formula No.2 as: QT (per millisecond)+1.75(Heart rate-60) .

**Table 2:** Prolonged§ QTc1¶ and QTc2 interval per lead in case (n=37) and control group (n=37)

**Leads**	**Length of QT**	**QTc**_1_ **,** **mean±SD** **(per milisecond)**			**QTc**_2_ **, ** **mean±SD** **(per milisecond)**		
		**Case **	**Control **	**P-value** *	**Case**	**Control **	**P-value** *
I	Normal	30 (81.1%)	37 (100%)	0.005	36 (97.3%)	37 (100%)	0.314
II	Prolonged	7 (18.9%)	-		1 (2.7%)	-	
III	Normal	25(67.6%)	37(100%)	0.000	33(89%)	37(100%)	0.040
aVR	Prolonged	12(32.47)	-		4(11%)	-	
aVL	Normal	28(75.7%)	37(100%)	0.001	34(91%)	37(100%)	0.077
aVF	Prolonged	9(24.3%)	-		3(9%)	-	
V1	Normal	25(67.6%)	36(97.3)	0.001	32(86%)	37(100%)	0.021
V2	Prolonged	12(92.3%)	1(2.7%)		5(14%)	-	
V3	Normal	29(78.4%)	37(100%)	0.003	36(97%)	37(100%)	0.314
V4	Prolonged	8(21.6%)	-		1(3%)	-	
V5	Normal	29(78.4%)	37(100%)	0.003	33(89%)	37(100%)	0.040
V6	Prolonged	8(21.6%)	-		4(11%)	-	

* P-value<0.05 is statistically significant.

¶ QTc_1_ is corrected QT interval that calculated by formula No: 1as: QT/√RR . QTc_2_ is corrected QT interval that calculated by formula No:2 as: QTc_2_=QT(per millisecond)+1.75(Heart rate-60)

§ QTc_1_ and QTc_2_ higher than 0.450 second for females and higher than 0.440 second in males were considered prolonged.

## Discussion

In the present study, we assessed correlation between corrected QT interval prolongation and severity of cirrhosis based on Child Classification in each ECG lead. Results showed that mean of QTc_1_ and QTc_2_ were longer in cirrhotics than control group. There was a significant correlation between Child score and length of QTc_1_ in leads: III, AVL, V2, V6. Besides, there were significant differences in length of QTc_1_ in leads: V3 and V6; and QTc_2_ in lead V3 between Child Classification.

In a study done in 2003 by Midis et al., 52 cirrhotic patients were entered into a study where the severity of cirrhosis was scored by Child-Pugh classification (9). In that study, QTc was longer in cirrhotic group as compared to the control group (0.471 second, P=0.0007 and 0.461 second, P=0.0017) and prolongation of QTc in cirrhotic patients had a correlation with the Child B and C classification which was statistically significant (0.489 second, p=0.001 and 0.480 second, p=0.0002) but not so in child A classification in cirrhotics (0.445 second, p=0.4366). In another study done by Patel D et al. in 2014, a number of 51 cirrhotic patients were without heart disease. In this study, prolonged QT was associated with worse liver function (higher INR, lower albumin) (10). 

In the study done in Romania in 2011 (11), there was a statistically significant correlation with the QTc and Child Classification. QTc was significantly longer in class C patients (520±45 milliseconds) compared with those in class "A" in cirrhotics (462±25 milliseconds; p=0.027). In study by Marafioti V et al. in 2015, QT interval prolongation was strongly associated with hepathic encephalopathy (12). In previous studies done on the correlation between QT interval prolongation and cirrhosis, only Bazett's formula was used. This formula has a low accuracy.More than 30% of normal ECGs have been reported as having prolonged QT with this formula.This is because it overcorrects the QT interval in low heart rate and undercorrects it in higher heart rates (13). In our study, we corrected QT interval with 2 formulas; first, the Bazette formula as: QTc=QT/√RR and second formula as: QTc=QT+1.75 (heart rate-60) (per millisecond). The latter formula is the newest formula recommended by Joint Committee of Professional Organization. This formula is a linear formula which is less affected by patient's heart rate (13). Therefore, in our opinion, the results of present study are more accurate than previous studies about corrected QT interval prolongation and cirrhosis.

The current study showed that not only cirrhosis can induce QT interval prolongation but also in lead V3, the prolongation of QT interval directly correlates with the severity of cirrhosis based on Child classification using both formulas (QTc_1_: P=0.031, QTc_2_: P=0.039).

Unlike in general population and patients with ischemic heart disease, the pathophysiology and complication of prolonged QT in cirrhotic patients is poorly understood. It may turn out that, cirrhotics may be more resistant to complication of QT interval prolongation by unknown mechanism in comparison with general population and patients with ischemic heart disease, although in a study by Kim SM et al. in 2017, QT interval prolongation in cirrhotic patients increased the risk of mortality (14).

Until further studies are done, there should be efforts to avoid factors which would contribute to this prolongation. For example, fluoroquinolones are used widely for urinary tract infection (UTI) and prophylaxis of spontaneous bacterial peritonitis (SBP) in cirrhotics can induce QT interval prolongation; therefore, these medications should be used with caution if absolutely indicated. Likewise, electrolyte imbalance like hypokalemia, hypocalcemia and hypomagnesemia which could result to diuretics and malnutrition should be vigorously corrected. We believe that more attention should be given to QT interval prolongation at pre, pri, and postoperative care of cirrhotics undergoing liver transplantation. Hence during cardiovascular assessment, to determine the cardiac risk of liver transplantation, physicians should pay more attention to QT interval prolongation at pre-,peri- and postoperative care of cirrhotic patients.

This study has several limitations. First, it was basically a cross-sectional study, and unfortunately, we did not access to ECG before cirrhosis and after liver transplantation. As a result, we could not evaluate the degree of QT lengthening in correlation with cirrhosis and liver transplantation. Second, besides the severity of cirrhosis, the etiology of cirrhosis may influence the length of QT interval. This however was not evaluated in our study. In total, cirrhosis can induce QTc interval prolongation. In Patel D et al.’s study in 2014, besides the other known factors affecting repolarization, cirrhosis can affect ventricular repolarization and induced longed QT (10). In another study by Young Huh et al. in 2014, QT interval in child class C patients was longer than child class A patients (15). Lead V3 has statistically significant correlation with the severity of cirrhosis based on child classification in both formulas used. (QTc_1_: p=0.031, QTc_2_: p=0.039). Perhaps, QT interval prolongation can be a prognostic factor in more advanced cirrhosis with higher risk. As a consequence, we propose that QT interval prolongation be added as a criterion for prioritizing liver transplantation.

In conclusion, cirrhosis can induce QTc interval prolongation. Lead V3 has statistically significant correlation with the severity of cirrhosis based on Child Classification. 
